# D-amino acid oxidase is expressed in the ventral tegmental area and modulates cortical dopamine

**DOI:** 10.3389/fnsyn.2014.00011

**Published:** 2014-05-02

**Authors:** Jill F. Betts, Judith V. Schweimer, Katherine E. Burnham, Philip W. J. Burnet, Trevor Sharp, Paul J. Harrison

**Affiliations:** ^1^Department of Psychiatry, University of OxfordOxford, UK; ^2^Department of Pharmacology, University of OxfordOxford, UK

**Keywords:** DAAO, DAO, D-serine, microdialysis, NMDA receptor, schizophrenia

## Abstract

D-amino acid oxidase (DAO, DAAO) degrades the NMDA receptor co-agonist D-serine, modulating D-serine levels and thence NMDA receptor function. DAO inhibitors are under development as a therapy for schizophrenia, a disorder involving both NMDA receptor and dopaminergic dysfunction. However, a direct role for DAO in dopamine regulation has not been demonstrated. Here, we address this question in two ways. First, using *in situ* hybridization and immunohistochemistry, we show that DAO mRNA and immunoreactivity are present in the ventral tegmental area (VTA) of the rat, in tyrosine hydroxylase (TH)-positive and -negative neurons, and in glial fibrillary acidic protein (GFAP)-immunoreactive astrocytes. Second, we show that injection into the VTA of sodium benzoate, a DAO inhibitor, increases frontal cortex extracellular dopamine, as measured by *in vivo* microdialysis and high performance liquid chromatography. Combining sodium benzoate and D-serine did not enhance this effect, and injection of D-serine alone affected dopamine metabolites but not dopamine. These data show that DAO is expressed in the VTA, and suggest that it impacts on the mesocortical dopamine system. The mechanism by which the observed effects occur, and the implications of these findings for schizophrenia therapy, require further study.

## Introduction

D-amino acid oxidase (DAO, DAAO; EC 1.4.3.3) is an enzyme which oxidatively deaminates neutral D-amino acids, including those derived from food and intestinal flora (Pollegioni et al., 2007). Its relevance for neurobiology emerged when one of its substrates, D-serine, was shown to be synthesized endogenously in brain, and to be a major co-agonist at the NMDA receptor (Schell et al., 1995; Wolosker et al., 1999; Mothet et al., 2000). Subsequent work has highlighted the roles of D-serine and its importance for brain function and dysfunction (Schell et al., 1997; Stevens et al., 2003; Yang et al., 2003; Shleper et al., 2005; Panatier et al., 2006; Strick et al., 2011; Fossat et al., 2012; Papouin et al., 2012; Gustafson et al., 2013; for review, see Wolosker et al., 2008; Oliet and Mothet, 2009; Van Horn et al., 2013), and it has become clear that DAO activity is a determinant of D-serine levels (Almond et al., 2006; Adage et al., 2008; Duplantier et al., 2009; Strick et al., 2011).

Interest has grown in the role of DAO in several neuropsychiatric disorders, especially schizophrenia, a disorder in which NMDA receptor-related hypofunction is pathophysiologically implicated (Javitt and Zukin, 1991; Olney et al., 1999; Tsai and Coyle, 2002; Kantrowitz and Javitt, 2010; Marek et al., 2010; Coyle, 2012) and is a target of much therapeutic research (Krystal et al., 2010; Moghaddam and Javitt, 2012). Evidence that DAO contributes to the glutamatergic dysfunction in schizophrenia is threefold (Verrall et al., 2010; Labrie et al., 2012; Sacchi et al., 2013): it may be a susceptibility gene (Chumakov et al., 2002; Allen et al., 2008; Shi et al., 2008; Sun et al., 2008); DAO expression and activity are increased (Kapoor et al., 2006; Verrall et al., 2007; Burnet et al., 2008a; Madeira et al., 2008); and DAO inhibitors show preliminary preclinical (Adage et al., 2008) and clinical (Lane et al., 2013) evidence for therapeutic efficacy.

DAO is conventionally viewed as a hindbrain enzyme, with little evidence for functionality in other brain regions (Horiike et al., 1994). This is a puzzle with regard to schizophrenia, which is primarily a disorder of the cerebral cortex, and one in which aberrant dopaminergic function plays a key role in pathophysiology (Howes and Kapur, 2009; Laruelle, 2013) and treatment (Kapur and Mamo, 2003). To date, little is known about whether and how DAO impacts on the dopamine system. Moreno et al. (1999) reported DAO immunoreactivity in neurons and glia of the rat substantia nigra and ventral tegmental area (VTA), and Verrall et al. (2007) found initial evidence for DAO immunoreactivity in neurons of human substantia nigra. Fernandez-Espejo et al. (2008) showed that injection of the prototypical DAO inhibitor sodium benzoate (Klein and Kamin, 1941; van den Berghe-Snorek and Stankovich, 1985) into the rat VTA affected cocaine sensitization (a dopamine-mediated behavior), but no neurochemical characterization was reported. Finally, ddY/DAO^−^ mice, which express inactive DAO due to a point mutation in the gene (Gly^181^Arg), show differential responses to amphetamine compared to wild-type mice (Hashimoto et al., 2008). Despite these studies, neither the presence of DAO in the VTA, nor an effect of DAO on dopamine neurons, has yet been clearly established.

The present study had two objectives. First, to determine whether or not DAO mRNA and immunoreactivity are present in the rat VTA, using *in situ* hybridization and immunohistochemistry. Second, to assess whether VTA DAO impacts on the mesocortical dopamine projection, by measuring cortical dopamine using *in vivo* microdialysis after acute inhibition of VTA DAO with sodium benzoate. In addition, since the effects of DAO inhibition are often assumed to be exerted via the resulting elevation of D-serine availability, the effects on cortical dopamine of intra-VTA injection of D-serine were also studied, with or without sodium benzoate. Our results show that DAO mRNA and protein are present in the VTA, in neurons and glia, and that intra-VTA injection of a DAO inhibitor acutely increases levels of cortical dopamine and its metabolites. However, the effect does not appear to be mediated entirely via D-serine, and the mechanism remains unclear.

## Materials and methods

### *In situ* hybridization histochemistry

To detect and localize DAO mRNA in the VTA, we used *in situ* hybridization histochemistry. 10–15 coronal sections (14 μm) through the VTA, or cerebellum (used as a positive control), were cut on a cryostat from four fresh frozen adult Sprague-Dawley rat brains, collected onto polylysine-coated slides and stored at −80°C. Before use, sections were fixed in 4% formaldehyde (in diethylpyrocarbonate [DEPC]-treated PBS) before being treated with DEPC-treated triethanolamine containing 0.25% acetic anhydride, dehydrated in graded ethanols and chloroform (5 min each), rehydrated to 95% ethanol and air-dried.

DAO cDNA was amplified from rat cerebellar cDNA using forward and reverse primers (forward sequence: GTGATGCGCGTGGCCGTGAT; reverse sequence: GGAATACACCTCCGAGTGTA), purified and ligated into pGEM-T Easy Vector. Plasmids were transformed into *E. Coli*, grown up overnight and positive colonies selected using blue-white reagent. Plasmids were extracted and purified using ChargeSwitch^®^-Pro Plasmid Miniprep Kit and eluted with 50 μl elution buffer. 10 μg plasmid was then digested with either SacII or PstI to linearize the construct.

To create the riboprobe, approximately 1 μg of linearized plasmid was dried down with [^35^S]UTP and then incubated with NTPs, RNAsin, reaction buffer, dithiothreitol (DTT) and either SP6 or T7 RNA polymerase to transcribe the SacII and PstI linearized constructs, producing sense, or antisense sequences, respectively. The plasmid DNA template was then removed using DNase. The probe was hydrolyzed by adding hydrolysis buffer and tRNA and incubating at 60°C for 34 min. The hydrolyzed probe was purified using NICK columns (GE Healthcare). The probe was diluted to 1.2 × 10^4^cpm/μl in hybridization buffer containing DTT and 200 μl added to each section. Sections were cover-slipped and incubated overnight at 45°C in trays prepared with filter paper soaked in 4× saline-sodium citrate buffer (SSC) containing 50% formamide. Sections were rinsed twice in 2× SSC at room temperature before sequential stringency washes were carried out to remove adventitiously bound probe: RNase A buffer, 30 min at room temperature; 2× SSC, 10 min at 55°C; 0.5× SSC, 10 min at 55°C; 0.1× SSC, 3 × 20 min at 55°C; 0.1× SSC, 45 min at room temperature. Sections were rinsed in dH_2_0, dipped in autoradiography emulsion (Amersham, UK), and exposed for 2 months at 4°C. Sections were developed, counterstained using cresyl violet, and cover-slipped.

### Northern blotting

The DAO riboprobe was verified by northern blotting, performed using standard methods. Briefly, total cellular RNA (20 μg) was prepared from cerebellum using the Tri-reagent (Sigma-Aldrich, UK), denatured and electrophoresed through 1.2% formamide-agarose gels and transferred to a nylon membrane. The membrane was pre-hybridized for 1 h at 42°C in hybridization buffer (5× saline-sodium citrate (SSC), 5× Denhardt's solution, 1% sodium dodecyl sulfate (SDS), 50% formamide, and 100 μg/ml of denatured salmon sperm DNA). The full length rat DAO open reading frame (1042 bp) was labeled with [^32^P]-CTP using random primers and the Klenow fragment of DNA polymerase I. The cDNA probe was purified, denatured at 95°C, and mixed with fresh hybridization buffer. This was incubated with the membrane overnight at 42°C. The membrane was then washed in 2× SSC (10 min), 0.5× SSC (10 min), and twice in 0.1× SSC (30 min) at 55°C. All solutions contained 0.1% SDS. The membrane was exposed to Kodak XAR-5 film at −70°C with intensifying screens.

### Immunofluorescence

To investigate whether DAO protein as well as DAO mRNA is expressed in VTA, we used immunofluorescence with an anti-DAO antibody. The antibody was a rabbit polyclonal, directed against a C-terminal peptide sequence (H-CGRILEEKKLSRMPPSHL-OH; N-terminal Cys added for coupling); the antibody has been validated by western blotting in rat brain previously (Verrall et al., 2007) and staining is abolished in DAO knockout mice (Schweimer et al., in review). We co-immunostained sections for tyrosine hydroxylase (TH) to identify VTA dopaminergic neurons, and for glial fibrillary acidic protein (GFAP) to label astrocytes.

Adult Sprague-Dawley rats (*n* = 3) were perfused using 4% paraformaldehyde and the brains removed and cryoprotected in sucrose solution. 20 μm sections containing VTA or cerebellum were cut using a cryostat, washed in PBS, then incubated in 50 mM ammonium chloride for 10 min. Further washing was carried out once in PBS, and twice in PBS containing Triton X-100 at 0.2% (PBSX), before blocking for 1 h in 6% normal donkey serum in PBSX. VTA sections (*n* = 6 per rat) were incubated overnight at 4°C with the anti-DAO antibody at 1:500 in 2% normal donkey serum in PBSX, with chicken primary anti-TH antibody (Abcam ab76422) at 1:1000 and goat primary anti-GFAP antibody (Abcam ab53554) at 1:2000. Following washes in PBS, VTA sections were soaked for 1 h in secondary donkey anti-rabbit IgG at 1:1000 (Alexa Fluor^®^ 488, A-21206, Invitrogen), donkey anti-chicken IgG at 1:1000 (Dylight 405, 703-475-155, Jackson Immunoresearch) and donkey anti-goat IgG at 1:1000 (Cy3, 705-166-147, Jackson Immunoresearch). Sections were then washed, once in PBSX, once in PBS and once in PB (saline), mounted onto slides, and coverslipped using Vectashield mountant. Cerebellar sections were co-immunostained for DAO and GFAP in the same way, but the anti-TH antibody was not used.

### *In vivo* microdialysis and high performance liquid chromatography

*In vivo* microdialysis, with HPLC detection, was used to measure extracellular dopamine and its metabolites homovanillic acid (HVA) and 3,4-dihydroxyphenylacetic acid (DOPAC) in the medial frontal cortex of anaesthetized rats following intra-VTA injection of sodium benzoate, D-serine, the combination, or vehicle. All animal procedures were carried out in accordance with the UK Animals (Scientific Procedures) Act 1986 and associated Home Office guidelines, and with local ethical approval.

Adult male Sprague-Dawley rats (Harlan, UK) were anaesthetized with chloral hydrate (500 mg/kg i.p.) and mounted in a stereotaxic frame in the flat skull position. Anesthesia was maintained with supplementary doses of chloral hydrate, and hydration sustained using 4% glucose in 0.18% saline. A craniotomy was made using a drill (Foredom^®^, Bethel, USA) and a microdialysis probe (crafted in-house) was stereotaxially implanted into the medial frontal cortex (AP +3.2 mm; ML +0.6 mm; DV −5.0 mm, relative to bregma and dura; Paxinos and Watson, 1998). The probe was secured using a cranial screw (2 mm, Royem Scientific, Luton, UK) and Simplex Rapid™ dental cement (Kemdent^®^, Swindon, UK). The probe was perfused with artificial cerebrospinal fluid (containing 140.0 mM NaCl, 3.0 mM KCl, 1.2 mM Na_2_HPO_4_.2H_2_O, 0.27 mM NaH_2_PO_4_.H_2_O, 1.0 mM MgCl_2_.6H_2_O, 2.4 mM CaCl_2_, and 7.2 mM glucose) containing 3 μM nomifensine at a flow rate of 1 μl/min. Dialysates were collected every 20 min in Eppendorf tubes containing 5 μl of 0.1 M perchloric acid. A guide cannula (26 G, 11 m length, PlasticsOne^®^) was implanted above the VTA (ML + 0.8 mm; AP −5.7 mm; DV −6.8 mm relative to bregma and dura). Following a stabilization period of at least 1 h, one of the following solutions was administered into the VTA [0.5 μl at a rate of 90 nl/min via injection needle (33G, PlasticsOne^®^)]: 200 μg/μl sodium benzoate (*n* = 4); 5 mM D-serine (*n* = 4); 5 mM D-serine plus 200 μg/μl sodium benzoate (*n* = 7); or saline (0.9% NaCl) vehicle (*n* = 6). Sampling continued for 2 h post-injection, after which pontamine sky blue (0.5 μl) was injected into the VTA for histological determination of injection site placement. Probe and injection needle placements were checked histologically.

Dialysate samples (25 μl) were analyzed for dopamine, DOPAC, and HVA, using HPLC with electrochemical detection. Analytes were separated on a Dynamax microsorb column (100 × 4.6 mm, 100-3 C18, Varian Inc., Middelburg, The Netherlands) using a mobile phase containing 15% methanol, 0.12 M NaH_2_PO_4_.H2O, 0.002 M NaCl, 0.1 mM EDTA, and 0.5 mM 1-octanesulphonic acid sodium salt (overall pH 4.17) at a flow rate of 1 ml/min (PU-1585, HPLC Pump, JASCO, Essex, UK). Assays were calibrated to standard solutions. JASCO ChromPass Chromatography Data System software (JASCO, Essex, UK) was used to plot the chromatograms and analyze the data. Analyte peaks were measured and quantified when the signal to noise ratio exceeded 2.

### Data analysis

Statistical analyses were carried out with SPSS for Windows 17. Repeated-measures ANOVAs were performed to assess the effects of time and drug on dopamine, DOPAC, and HVA relative to baseline. Simple main effects tests were carried out if the ANOVA was significant (*P* < 0.05).

## Results

### DAO mRNA is expressed in the VTA

**Figure 1:**
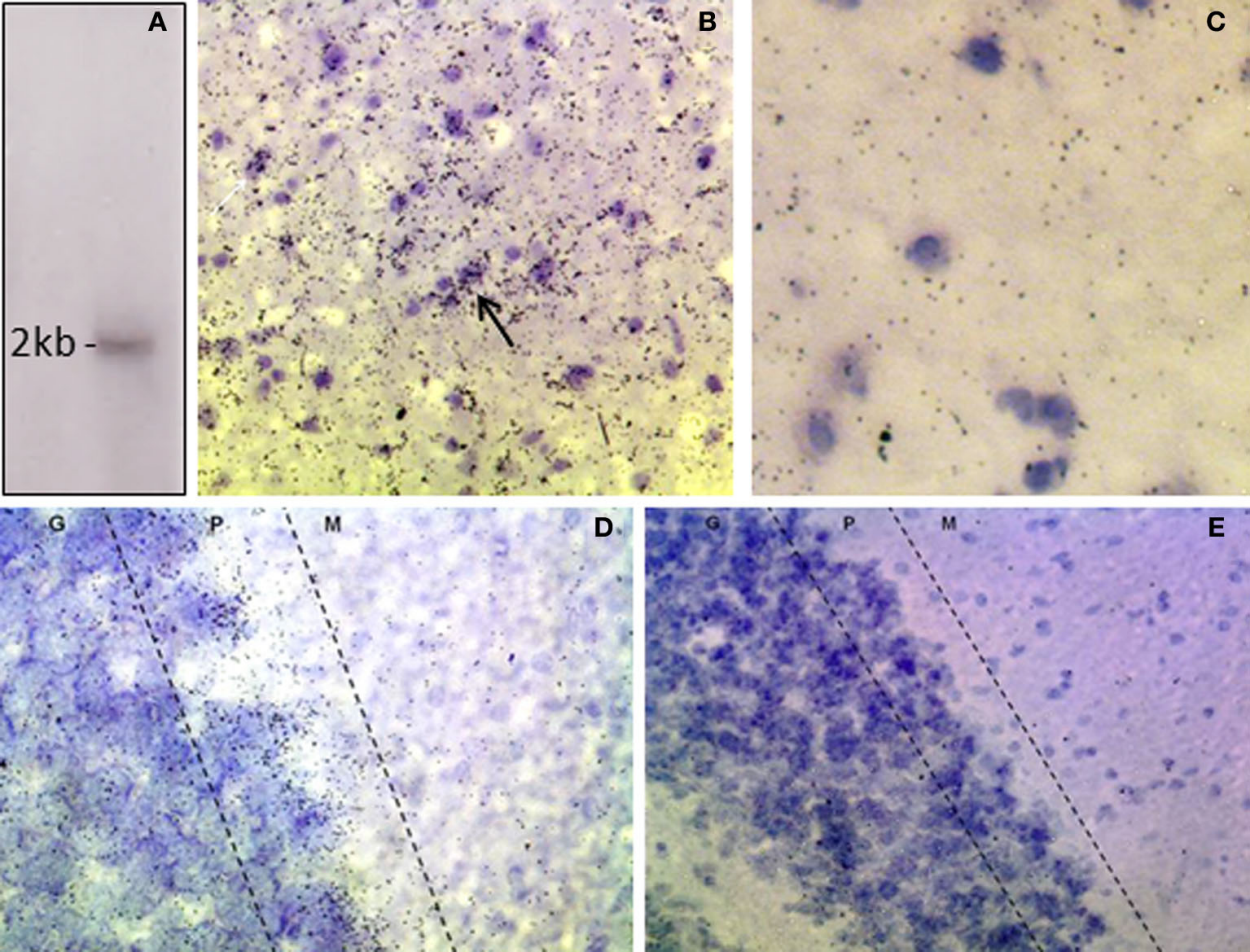
**Expression of DAO mRNA in rat VTA and cerebellum. (A)** Northern blot of rat cerebellar RNA showing that the DAO cRNA probe detects a single band of ~2 kb. **(B)** *In situ* hybridization, showing signal for DAO mRNA in the VTA, with clusters of grains over neurons (arrow). **(C)** VTA, sense strand hybridization control, showing low level of background signal. **(D)** Cerebellum, showing DAO mRNA concentrated in the Purkinje cell layer (P) with moderate signal in the granule cell layer (G) and low signal in the molecular layer (M). **(E)** Cerebellum, sense strand hybridization control. Sections are counterstained with cresyl violet.

The riboprobe detected a single band of the predicted size of the rat DAO transcript, ~2 kilobases (Figure 1A; Konno, 1998). *In situ* hybridization histochemistry showed expression of DAO mRNA in the rat VTA, with clustering over neurons and over the neuropil (Figure 1B). The latter is likely to reflect DAO mRNA in un-counterstained glial processes, since sense strand hybridization produced minimal background neuropil signal (Figure 1C). In the cerebellum, grains were concentrated in the Purkinje cell layer, as anticipated given the known localization of DAO to Bergmann glia which surround the Purkinje cells (Figure 1D). Cerebellar non-specific signal was low, as in VTA (Figure 1E).

### DAO immunoreactivity is present in the VTA

**Figure 2:**
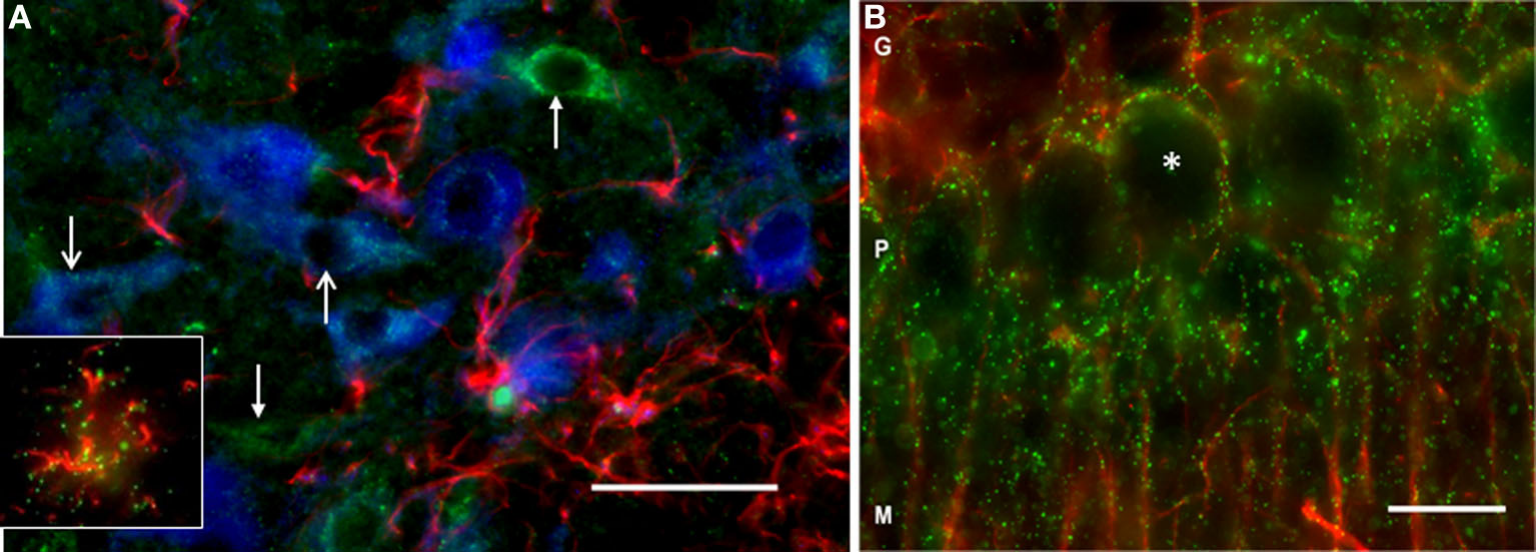
**DAO immunoreactivity in rat VTA and cerebellum. (A)** Triple-labeling in VTA showing immunofluorescence for DAO (green), tyrosine hydroxylase (TH, blue), and glial fibrillary acidic protein (GFAP, red). The open head arrows point to two TH-positive neurons immunoreactive for DAO; the closed head arrows show DAO-immunoreactive TH-negative neurons. The inset shows a DAO-immunoreactive GFAP-positive astrocyte. **(B)** Double-labeling in cerebellum, showing DAO (green), and GFAP (red) immunoreactivity. Punctate labeling for DAO is concentrated in the Purkinje cell layer (P), wherein it surrounds the unlabeled Purkinje cell somata (asterisk), and the immunoreactivity extends into the molecular layer (M) along GFAP-positive processes. G: granule cell layer. Bar: 50 microns.

Using immunofluorescence histochemistry, DAO immunoreactivity could be seen in VTA neurons and glia (Figure 2A). Staining was punctate within the cytoplasm, and with an absence of nuclear labeling. Many DAO-immunoreactive neurons were also TH-immunopositive; however, not all DAO-positive neurons were TH-immunopositive, and vice versa. DAO-immunoreactive GFAP-positive astrocytes were also identified (Figure 2A, insert). Thus, in VTA, DAO is detectable in some neurons, both dopaminergic and non-dopaminergic, and in glia. No quantification of immunolabeling was carried out. In the cerebellum, DAO immunostaining was visible around the periphery of Purkinje cells (but not over the cell bodies), and also in cell processes projecting into the molecular layer (Figure 2B), co-localizing with GFAP immunoreactivity, consistent with the known cerebellar localization of DAO in Bergmann glia.

### Effects of sodium benzoate, D-serine, and the combination, on extracellular dopamine levels in medial frontal cortex

**Figure 3:**
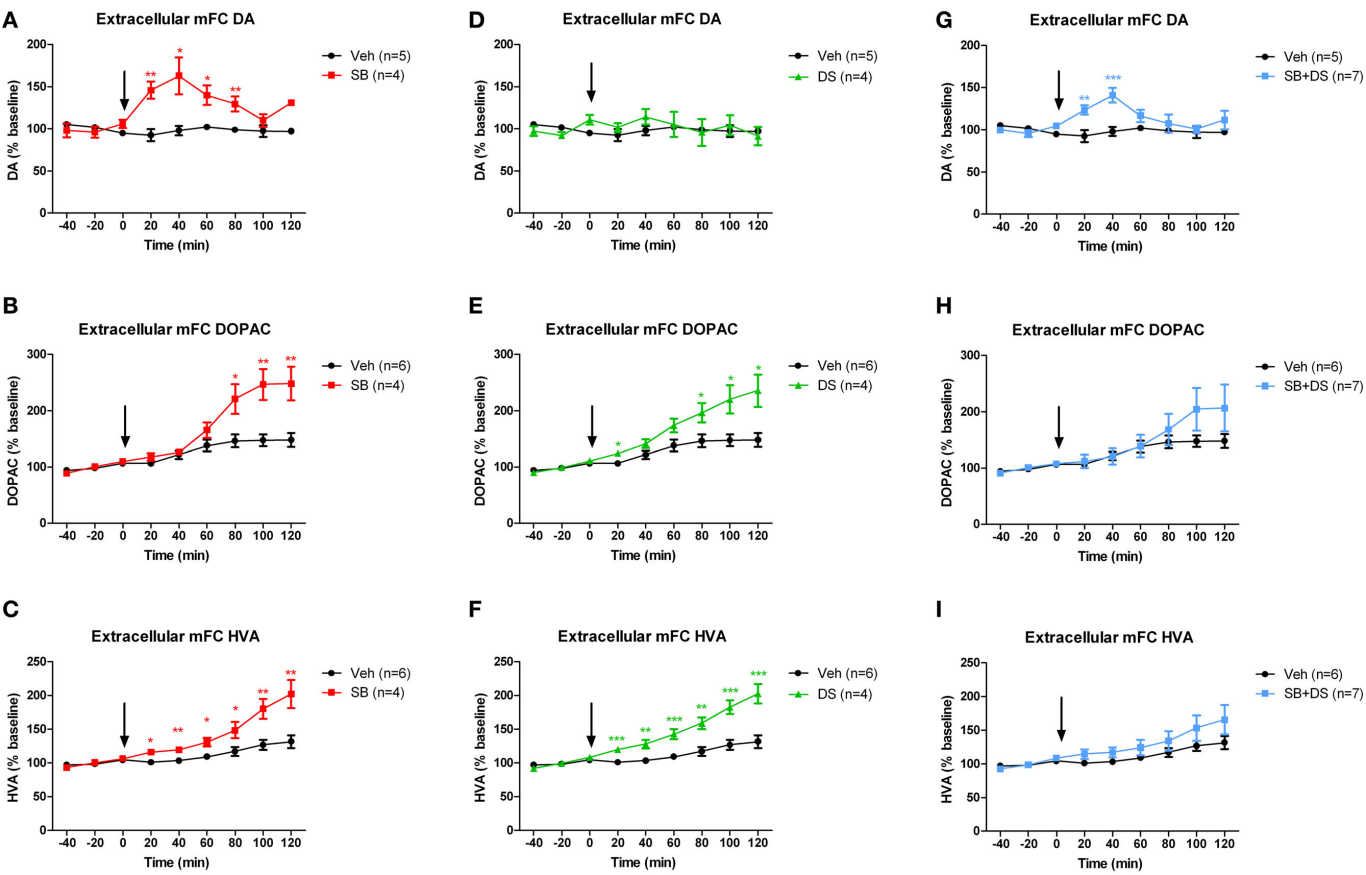
**Dopamine (DA) and its metabolites DOPAC and HVA in medial frontal cortex (mPFC) after injection of sodium benzoate (SB), D-serine (DS), the combination (SB+DS), or vehicle (Veh), into the rat VTA. (A–C)** Effects of sodium benzoate, showing increases in DA **(A)**, DOPAC **(B)**, and HVA **(C)**. **(D–F)** Effects of D-serine, showing no effect on DA **(D)**, but increases in DOPAC **(E)** and HVA **(F)**. **(G–I)** Effects of combined sodium benzoate and D-serine, showing increases in DA **(G)** and no significant change in DOPAC **(H)** or HVA **(I)**. The arrow in each panel denotes the time of injection. ^*^p < 0.05; ^**^p < 0.01; ^***^p < 0.005.

Sodium benzoate, injected into the VTA, increased extracellular dopamine in the medial frontal cortex [*F*_(1, 7)_ = 14.559, *p* = 0.007], peaking at 40 min post-injection [main effect of time: *F*_(5, 35)_ = 5.413, *p* = 0.001; drug × time interaction *F*_(5, 35)_ = 5.456, *p* = 0.001; Figure 3A]. Levels of extracellular DOPAC (Figure 3B) and HVA (Figure 3C) were also elevated after sodium benzoate, and peaked at 100 and 120 min post-injection, respectively [DOPAC: effect of drug *F*_(1, 8)_ = 8.643, *p* = 0.019; effect of time *F*_(5, 40)_ = 48.919, *p* < 0.001; drug × time interaction *F*_(5, 40)_ = 16.775, *p* < 0.001. HVA: effect of drug *F*_(1, 8)_ = 11.931, *p* = 0.009; effect of time *F*_(5, 40)_ = 37.848, *p* < 0.001; drug × time interaction *F*_(5, 40)_ = 8.625, *p* < 0.001].

We next investigated whether intra-VTA injection of D-serine would have the same effect as sodium benzoate. D-serine had no effect on dopamine levels in medial frontal cortex when compared with vehicle [effect of drug *F*_(1, 7)_ = 0.223, *p* = 0.651; effect of time *F*_(5, 35)_ = 1.081, *p* = 0.388; drug × time interaction *F*_(5, 35)_ = 0.854, *p* = 0.521; Figure 3D]. However, D-serine did increase DOPAC (Figure 3E) and HVA (Figure 3F), both of which continued to rise throughout the 120 min after injection [DOPAC: effect of drug *F*_(1, 8)_ = 8.582, *p* = 0.019; effect of time *F*_(5, 40)_ = 30.886, *p* < 0.001; drug × time interaction *F*_(5, 40)_ = 7.006, *p* < 0.001; HVA: effect of drug *F*_(1, 8)_ = 22.805, *p* = 0.001; effect of time *F*_(5, 40)_ = 41.256, *p* < 0.001; drug × time interaction *F*_(5, 40)_ = 8.076, *p* < 0.001].

Finally, we assessed whether the combination of sodium benzoate and D-serine would show a greater effect than either alone. Concurrent intra-VTA injection of sodium benzoate and D-serine increased extracellular dopamine in medial frontal cortex, peaking 40 min post-injection [effect of drug *F*_(1, 10)_ = 5.504, *p* = 0.041; effect of time *F*_(5, 50)_ = 3.252, *p* = 0.013; drug × time interaction *F*_(5, 50)_ = 3.678, *p* = 0.007; Figure 3G]. There was an equivocal effect on DOPAC (Figure 3H), with no main effect of drug [*F*_(1, 11)_ = 0.714, *p* = 0.416], but a significant effect of time [*F*_(5, 55)_ = 13.062, *p* < 0.001], and a drug × time interaction [*F*_(5, 55)_ = 3.117, *p* = 0.015]. HVA was not significantly affected by the sodium benzoate/D-serine combination (Figure 3I), with no main effect of drug [*F*_(1, 11)_ = 1.764, *p* = 0.211], and no drug × time interaction [*F*_(5, 55)_ = 1.081, *p* = 0.381].

## Discussion

Little is known as to the interaction between DAO and the dopamine system. We investigated the expression and localization of DAO in the rat VTA, and carried out a preliminary study of its functional impact upon the mesocortical dopamine system. We found that DAO mRNA (Figure 1) and protein (Figure 2) are present in the VTA, as demonstrated using *in situ* hybridization and immunofluorescence, respectively. DAO expression was identified in three cell types: TH-positive neurons, TH-negative neurons, and GFAP-positive astrocytes. *In vivo* microdialysis studies (Figure 3) showed elevated levels of dopamine and its metabolites in medial frontal cortex after intra-VTA administration of the DAO inhibitor sodium benzoate. Co-administration of sodium benzoate with D-serine did not enhance this effect, and D-serine alone did not alter dopamine but did increase DOPAC and HVA. These results show the presence of DAO in the VTA, suggest a physiological role for DAO within VTA in modulating cortical dopamine, and may have implications for use of DAO inhibitors in schizophrenia.

### Expression of DAO in the VTA

DAO mRNA and immunoreactivity were both detected in the rat VTA, extending existing studies which suggested expression of DAO in the dopaminergic midbrain (Moreno et al., 1999; Verrall et al., 2007).

DAO is classically considered to be a glial enzyme, and our findings in the cerebellum are consistent with many previous studies which show DAO to be enriched primarily in Bergmann glia (Weimar and Neims, 1977; Horiike et al., 1987; Verrall et al., 2007; Ono et al., 2009; Burnet et al., 2011). In the VTA, DAO was also glial, being seen in some GFAP-immunoreactive astrocytes. However, we also found DAO mRNA and immunoreactivity in VTA neurons, the latter being TH-positive (presumed dopaminergic) neurons, as well as some TH-negative neurons. The presence of neuronal DAO in other brain regions has been reported in some previous studies (Moreno et al., 1999; Verrall et al., 2007: Popiolek et al., 2011), and recently in spinal cord (Paul et al., 2014). In glia, DAO is localized to peroxisomes (Sacchi et al., 2008), but in neurons it has a broader subcellular distribution and associates with the presynaptic protein Bassoon, an interaction which inhibits DAO activity (Popiolek et al., 2011). Whether this also applies to VTA neurons, and whether this differential subcellular distribution is paralleled by functional differences between neuronal and glial DAO is unknown. Further studies are also needed to establish the proportions and neurochemical identities of DAO-positive cells in the VTA (e.g., using quantitative immunohistochemistry, and single cell PCR).

### Inhibition of VTA DAO affects cortical dopamine

A role for DAO in the VTA cannot be assumed just by virtue of its expression therein, since DAO mRNA and immunoreactivity are also detected in frontal cortex (Moreno et al., 1999; Hashimoto et al., 2007; Verrall et al., 2007), yet there is limited evidence for functionality of cortical DAO, and cortical D-serine concentrations are not elevated in the ddY/DAO^−^ mouse (Yamanaka et al., 2012). Our *in vivo* microdialysis data suggest that VTA DAO is indeed functional (complementing the observations of Fernandez-Espejo et al., 2008), since local injection of the DAO inhibitor sodium benzoate increased extracellular dopamine and its metabolites in medial frontal cortex (Figures 3A–C). The increase in dopamine was rapid and transient, whereas the elevation in HVA and DOPAC was delayed and longer lasting. This is in keeping with earlier findings that the response of dopamine metabolites to stimulation of dopamine neurons is delayed compared to that of dopamine itself (Nakahara et al., 1992); the likely explanation is that the metabolites arise principally from newly synthesized dopamine, and that dopamine synthesis is activated when the firing of the neurons is elevated (Zetterström et al., 1988).

A plausible mechanism for the observed effect of VTA sodium benzoate on cortical dopamine is as follows. Sodium benzoate inhibits local DAO, decreasing D-serine degradation and thereby increasing extracellular D-serine concentrations; the increased co-agonist availability enhances NMDAR signaling, with a resulting increase in dopamine neuron firing and thence dopamine release. This explanation is consistent with the known metabolism of D-serine by DAO (Almond et al., 2006; Adage et al., 2008; Duplantier et al., 2009; Strick et al., 2011), the effects of D-serine on NMDA receptor function (Schell et al., 1997; Mothet et al., 2000; Stevens et al., 2003; Panatier et al., 2006; Fossat et al., 2012; Papouin et al., 2012), and the regulation of dopamine neuron firing and release by NMDA receptors. That is, activation of VTA NMDA receptors enhances dopamine neuron burst firing (Seutin et al., 1990; Chergui et al., 1993; Wang and French, 1993) and stimulates cortical dopamine release (Kalivas et al., 1989; Takahata and Moghaddam, 1998; Westerink et al., 1998). Our recent finding that DAO knockout mice show increased VTA dopamine neuron burst firing when compared with their wild-type littermates supports this model (Schweimer et al., in review). The affected population of VTA NMDA receptors likely resides on the dopamine neurons themselves (Petralia et al., 1994; Standaert et al., 1994), although indirect effects via NMDA receptors located on non-dopamine VTA neurons, or glia, cannot be excluded (Steffensen et al., 1998).

However, there are important caveats to this proposed sequence of events: we did not directly measure VTA DAO activity, nor levels of D-serine and other NMDA receptor co-agonists, nor indices of NMDA receptor signaling. As such, any explanation for the effect of sodium benzoate on cortical dopamine based on alterations in these parameters is speculative. Indeed, regarding the first caveat, the postulated mechanism of effect of DAO inhibition (via elevation of D-serine availability) is somewhat at odds with the fact that D-serine had no effect on dopamine (Figure 3D), and the combination of sodium benzoate plus D-serine did not elevate cortical dopamine more than sodium benzoate alone (Figures 3G vs. 3A). Moreover, the rise in dopamine metabolites, especially HVA, was less pronounced after combined sodium benzoate/D-serine injection (Figures 3H,I) than with either drug alone (Figures 3B,C,E,F), although the differences were not statistically different. Whilst the results remain preliminary, given the sample sizes, the findings do together suggest that sodium benzoate may be acting through a mechanism partly or wholly independent of DAO inhibition. We are not aware of any such off-target effects being reported (e.g., inhibition of other enzymes), but the metabolism of sodium benzoate does interact with availability of glycine, another NMDA receptor co-agonist (Van Hove et al., 2005), an interaction which may be relevant in some way. Potential non-DAO-mediated effects could be tested by (a) direct demonstration of VTA DAO inhibition after sodium benzoate administration using enzyme assay; (b) replicating the present results using additional DAO inhibitors, and (c) repeating the present study in DAO knockout or ddY/DAO^−^ mice and showing that cortical dopamine is unaffected by sodium benzoate.

Assuming that the present findings do result from DAO inhibition, the question remains as to why D-serine administration did not reproduce or synergize the effect of sodium benzoate on cortical dopamine. Perhaps DAO inhibition affects a substrate other than D-serine, for example D-alanine, which is a high affinity NMDA receptor co-agonist (Tanii et al., 1994) metabolized by DAO (Molla et al., 2006; Horio et al., 2009), and whose levels rise markedly in rodents lacking DAO (Yamanaka et al., 2012). Alternatively, the injected D-serine may be rapidly removed from the synapse via a transporter (Helboe et al., 2003; Rutter et al., 2007; Burnet et al., 2008b; Sikka et al., 2010), or metabolized by serine racemase (Foltyn et al., 2005; Strisovsky et al., 2005). The fact that D-serine given alone increased the levels of dopamine metabolites (Figures 3E,F) but not dopamine (Figure 3D) suggests that it had a mild and transient enhancing effect on dopamine neurons, but that breakdown of the newly synthesized dopamine resulted in no detectable increase in dopamine itself (Zetterström et al., 1988). To help distinguish between these explanations, it would be valuable to measure local, extracellular NMDA receptor co-agonist concentrations after DAO inhibition.

Finally, to confirm that the observed effect of sodium benzoate on cortical dopamine is mediated via enhanced VTA NMDA receptor signaling, electrophysiological measurements of NMDA receptor currents, and use of selective NMDA receptor antagonists would be required. The use of mice with selective inactivation of NMDA receptors on dopamine neurons (Zweifel et al., 2011; Wang et al., 2011) would also be of interest.

### Implications for schizophrenia therapy

The rationale for DAO inhibitors as a treatment for schizophrenia has come from their potential to increase D-serine availability, thus correcting the postulated NMDA receptor co-agonist deficiency (Marek et al., 2010; Verrall et al., 2010; Labrie et al., 2012). The case is supported by findings that patients have increased DAO activity and expression (Kapoor et al., 2006; Verrall et al., 2007; Burnet et al., 2008a; Madeira et al., 2008), complemented by equivocal evidence for lower D-serine levels (Hashimoto et al., 2003; Bendikov et al., 2007; Brouwer et al., 2013) and initial findings that novel DAO inhibitors show efficacy in preclinical models of schizophrenia (Adage et al., 2008) and, for sodium benzoate, in the disorder itself (Lane et al., 2013).

All existing licensed drug treatments for schizophrenia are dopamine D2 receptor antagonists, and the question arises whether DAO inhibitors may also work, at least partly, via dopaminergic effects. Our results suggest that this is possible, but there are several important considerations to bear in mind. Firstly, systemic administration of the drugs may well give a different result (Bennett and Gronier, 2007; Smith et al., 2009), since NMDA receptors in frontal cortex and nucleus accumbens have differing effects on dopamine release compared to those located in the midbrain (Imperato et al., 1990; Takahata and Moghaddam, 1998; Del Arco and Mora, 2001). Indirect effects could also occur; e.g., inhibition of DAO in the cerebellum might impact on the cerebellar regulation of cortical dopamine (Mittleman et al., 2008; Rogers et al., 2011). Secondly, it will be important to study chronic as well as acute administration, and a range of doses. Thirdly, rat DAO has some different enzymatic properties compared to human DAO (Sacchi et al., 2013), highlighting the possibility of species differences in the relationship between DAO and dopamine. Finally, the dopaminergic dysfunction of schizophrenia is complex, with excess striatal dopamine underlying positive symptoms, and hypofunction of the mesocortical dopamine projection contributing to cognitive/negative symptoms (Weinberger, 1987; Davis et al., 1991; Howes and Kapur, 2009; Laruelle, 2013). Simplistically, therefore, enhancement of cortical dopamine release by DAO inhibition might be therapeutic for the latter domains, but if a similar effect occurred in striatum it might exacerbate positive symptoms. It is therefore notable that NMDA receptors appear to play a lesser role in regulation of dopamine in nucleus accumbens than in frontal cortex (Kalivas et al., 1989; Karreman et al., 1996; Doherty and Gratton, 1997; Westerink et al., 1998; Mathe et al., 1999), providing the possibility that DAO inhibition might be able to enhance cortical dopamine function without significantly increasing striatal dopamine. This speculation remains to be empirically tested.

In summary, we show that DAO is present in neurons and glia of the rat VTA, and provide preliminary evidence that VTA DAO influences the mesocortical dopamine system, since injection of a DAO inhibitor into the VTA increased dopamine in the frontal cortex. As well as replication of the current findings, considerable further study is required to extend them and to clarify the underlying mechanisms. Nevertheless, the present findings draw attention to the possible interaction between DAO and the dopamine system, which has potential relevance for the ongoing development of DAO inhibitors to treat schizophrenia and other disorders.

### Conflict of interest statement

The authors declare that the research was conducted in the absence of any commercial or financial relationships that could be construed as a potential conflict of interest.
